# Medical and Surgical Strategies in Vulvar Paget Disease: Let’s Throw Some Light!

**DOI:** 10.3390/jpm13010100

**Published:** 2023-01-01

**Authors:** Luigi Della Corte, Valeria Cafasso, Carmine Conte, Lara Cuomo, Pierluigi Giampaolino, Giada Lavitola, Giuseppe Bifulco

**Affiliations:** 1 Department of Neuroscience, Reproductive Sciences and Dentistry, School of Medicine, University of Naples Federico II, 80131 Naples, Italy; 2 Department of Public Health, University of Naples Federico II, 80131 Naples, Italy; 3 Department of General Surgery and Medical-Surgical Specialties—Institute of Obstetrics and Ginecology, A.O.U. Policlinico Rodolico—San Marco, University of Catania, 95124 Catania, Italy

**Keywords:** extramammary Paget’s disease, vulvar Paget’s disease, noninvasive/invasive vulvar Paget’s disease, medical treatment, surgery

## Abstract

Background: Vulvar Paget’s disease (VPD) is defined as a neoplasm of epithelial origin, mostly in postmenopausal women. Due to the extreme rarity of VPD, limited data about recommended treatment options are available. Surgical excision has been the treatment of choice although in the recent decade medical treatments have been proposed. Methods: A systematic computerized search of the literature was performed in the main electronic databases (MEDLINE, EMBASE, Web of Science, PubMed, and Cochrane Library), from 2003 to September 2022, in order to analyze all medical and surgical strategies used for the treatment of VPD. Results: Thirty-four articles were included in this review with findings as follows: 390 patients were treated with medical or other conservative treatment while 2802 patients were treated surgically; 235/434 (54%) patients had a complete response, 67/434 (15%) a partial response, 10/434 (2.3%) a stable disease, 3/434 (0.7%) disease progress, 3/434 (0.7%) died of the disease, 55/434 (13%) died of other causes during follow up while 7/434 (1.6%) had to stop topical treatments with 5% imiquimod cream because of side effects; 239/434 patients (55%) had a recurrence and 11/434 (2.5%) were lost to follow-up. The length of follow-up was variable, according to the different studies analyzed. Conclusion: VPD is a chronic disease with a high recurrence rate and low mortality. There are no significant differences in recurrence rates in patients who undergo surgery and those who do not and the margin status at the time of primary surgery and recurrence. Several surgical and medical approaches providing both local control of the disease and minimal tissue damage have been developed. Clock mapping, a recent preoperative vulvo-vaginal workup tool, can predict the invasiveness and the extension of VPD. However, to date, due to the different treatment options available and in the absence of a global consensus, it is critical to tailor treatments to individual patient characteristics and biopsy histopathologic findings, to ensure the best type of therapy.

## 1. Introduction

Vulvar Paget’s disease (VPD) is defined as a neoplasm of epithelial origin that usually develops in apocrine gland areas, and represents, in its invasive form, about 1–2% of vulvar neoplasms with metastatic potential and the most frequent location (60%) of extramammary Paget’s disease (EMPD) [1,2,3]. Postmenopausal Caucasian women (median age 72 years) are predominantly affected by EMPD [4]. EMPD can be classified according to the site of origin of the neoplasm in two forms: a primary EMPD, an intraepithelial adenocarcinoma originating in the epidermis that can evolve into an invasive carcinoma, and a secondary form that derives from metastatic diffusion to the skin [5]. Moreover, EMPD can precede or develop along with other malignant carcinomas. It is essential to carry out an accurate evaluation, in order to exclude neoplasms, such as breast examination, ileo-coloscopy, cystoscopy with urine cytology, serum tumor markers (Ca 125, CEA, CA 19.9, etc.) and computer tomography of the thorax, abdomen, and pelvis. Given its rarity, no clear epidemiological data are available, although the estimated incidence of EMPD in Europe is 0.7/100,000 per year [6].

Longstanding itching and vulvar pain or burning are the most common symptoms, although many patients may remain asymptomatic for several years and diagnostic de-lays are frequent [7]. The primary lesions appear as well-demarcated patchy erythematous or eczematous plaques, with frequent multifocal dyschromic and hyperkeratosis appearances [8]. Indeed, the classic ‘strawberries and cream’ description of vulvar Paget’s disease is represented by erythematous plaques with isolated islands of hyperkeratosis. Lesions are usually multifocal and can develop anywhere on the vulva, although they mainly occur in the labia majora, with the possibility of extension to the perineum, thighs, and mount pubis [9].

Surgery remains the cornerstone of therapy, but EMPD often spreads in an extended occult margin, beyond the apparent limits of the lesion, so that the visual clinical borders do not correspond to the histopathologic extent of the disease, necessitating repeated and sometimes mutilating operations [10]. However, the recurrence rate is high while surgery leads to anatomical, functional, and sexual morbidity. Furthermore, different topical therapies, such as laser ablation, imiquimod 5% cream, and radiation, have been described, as an alternative for patients who cannot undergo surgery.

The aim of this review is to present a wide and detailed description of all medical and surgical strategies used for the treatment of vulvar Paget’s disease.

## 2. Materials and Methods

The data research was conducted using the following databases: MEDLINE, EMBASE, Web of Sciences, Scopus, ClinicalTrial.gov, OVID, and Cochrane Library querying for all articles related to VPD from the inception of the database up to October 2022. The studies were identified with the use of a series of the following text words: extramammary Paget disease, vulvar Paget’s disease, noninvasive/invasive vulvar Paget’s disease, imiquimod, vulvectomy, vulvar cancer. The selection criteria of this narrative review included randomized clinical trials, nonrandomized controlled studies (observational prospective, retrospective cohort studies, case-control studies, case series), and review articles. A review of articles also included the abstracts of all references retrieved from the search. Conference papers and reviews and studies with information overlapping another publication were excluded. In the event of overlapping studies, we selected the most recent and/or most comprehensive manuscript.

**Figure 1:**
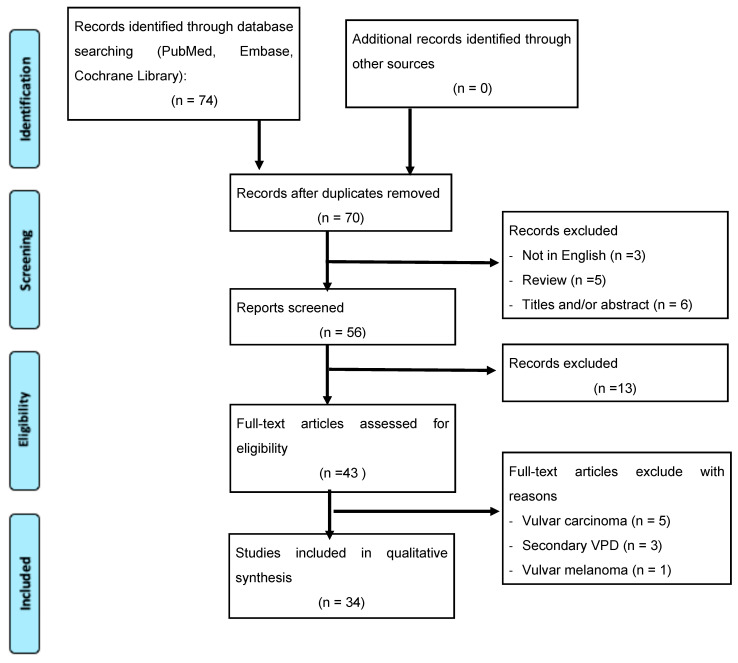
Flowchart of systematic review search.

We initially selected 74 studies from different databases; of these, only 70 records were screened. Of these records, 43 studies were assessed for eligibility whereas 4 were excluded because of being related to vulvar cancer or melanoma and 3 because they dealt with secondary vulvar Paget (Figure 1). Titles and/or abstracts of studies retrieved using the search strategy and those from additional sources were screened independently by 2 review authors (L.D.C. and V.C.) to identify studies that potentially met the aims of this review. The full text of these potentially eligible articles was retrieved and independently assessed by them. Any disagreement between them over the eligibility of particular articles was resolved through discussion with a third (external) collaborator (G.B.). Two authors (L.D.C. and V.C.) independently extracted data from articles about study features and included populations, types of intervention (medical therapy or surgery), and outcomes. Any discrepancies were identified and resolved through discussion (with a third external collaborator where necessary).

## 3. Results

Of the 34 articles included in this review, 10 were case reports [11,12,13,14,15,16,17,18,19,20], 10 case series (4 of them reporting 2 cases, 2 with 3 cases, 1 with 4 cases, 1 with 6 cases, 1 with 10 cases, and the last one reporting 44 cases) [21,22,23,24,25,26,27,28,29,30], 9 retrospective studies [31,32,33,34,35,36,37,38,39], 2 multicentric retrospective studies [40,41], 2 multicenter prospective studies [42,43] and 1 prospective study [44]. Women, 3244 in number, with primary or recurrent EMPD were included. The mean age was over 65 years (range 31–92) The youngest patient was 31 years old as reported by Nasioudis et al. [36] while the oldest was 92 years old [29,38,39,44]. The main type of vulvar lesion was a well-defined erythematous plaque, while the most complained symptom was itching, although soreness and burning were also quite frequent. The characteristics of the included patients are summarized in Table 1.

Regarding the type of treatment, 390 patients were treated with medical or other conservative treatments while 2802 patients were treated surgically; specifically, 182 patients analyzed were treated with topical 5% imiquimod cream [11,12,13,14,15,16,17,21,22,23,24,25,26,27,29,38,39,40,42,43], 72 with immunotherapy [36], 52 with radiotherapy [19,20,24,31,33,36], 32 with laser or other ablative methods [18,35,36,38,41,44], 23 patients with chemotherapy [14,36], 14 with topical 16% methyl aminolevulinate (MAL) + photodynamic therapy (PDT) [17,41], 10 with Fractional CO_2_ Laser irradiation followed by photodynamic therapy [44], 3 with 5-fluorouracil [35,37] and 2 patients underwent CO_2_ laser excision [34]. Among patients who underwent surgery, approximately 40% of them underwent local surgical excision [19,36,38], 31% simple partial/total vulvectomy [19,24,29,31,32,33,34,36,37,38], 7% radical vulvectomy [19,20,29,31,32,33,34,35,36,37,38], 7% total vulvectomy [38], 6% wide local excision [13,18,19,25,28,29,30,31,32,33,34,35,37,38], 1% hemivulvectomy [16,38], 0.2% and 0.1% skinning vulvectomy [22,34,35] and Mohs micrographic surgery (MMS), respectively [29,35]; moreover, about 5% of patients underwent inguinal lymphadenectomy [20,28,29,31,33,34] and 1% of them needed reconstructive surgery (V-Y plasty, transposition flap, rotational flap, skin graft) [19,20,22,28,29,30,34,36,37,38]. In about 3.6% of patients, the type of surgery was not specified [36]. For more details about treatments, see Table 2.

Of 516 patients, 70 (13.5%) experienced recurrence with a history of previous medical/surgical treatment, 446 patients received a first diagnosis of EMPD (86.4%). Out of a total of 434 patients, a complete clinical and histologic remission was observed in 235 patients (54%), 67 (15%) had partial response, 10 (2.3%) stable disease, 3 (0.7%) disease progression whereas 3 (0.7%) died because of disease. Moreover, 55 (13%) patients died of other causes not related to vulvar Paget during the follow up period while 7 patients (1.6%) discontinued topical therapy with 5% imiquimod cream because of side effects, mainly for intolerable local reactions and more rarely due to flu-like syndrome. Patients, 239 in number, (55%) had a recurrence and 11 (2.5%) were lost to follow up. The period of follow up for each individual case is shown in Table 3.

## 4. Discussion

Due to the extreme rarity of VPD, limited data about recommended treatment options are available. Currently, in cases of invasive VPD, surgical excision is indicated. Surgery can be a definitive and decisive treatment in VPD, particularly in the case of large, multifocal and invasive forms, even if it is burdened by postoperative complications, especially dehiscence. Indeed, surgery requires a hospital stay of variable duration, with a more or less rapid recovery, also depending on the type of surgery and on the individual characteristics of the patients.

Due to the tumor’s multifocal nature, irregular shape, and blurred contours, the resection margins are often positive and relapses are frequent [45,46]. In these circumstances as well as in the case of non-invasive patterns or poor general conditions, it is possible to choose conservative treatment as an alternative. Among these treatments, laser CO_2_ or photodynamic therapy, imiquimod cream, radiotherapy or chemotherapy may contribute to increased quality of life if compared with surgery, though with a lower success rate [46,47,48].

Imiquimod is considered the first-line treatment for anogenital warts, and it has been approved for the treatment of actinic keratosis and superficial basal cell carcinomas. It is used off-label as a therapy for different conditions such as vulvar, vaginal, and cervical intraepithelial neoplasia, EMPD, and skin metastases of malignant melanoma [49]. Data in the literature are heterogeneous, many studies described the use of imiquimod in the treatment of VPD due to possible application in elderly patients, in the case of comorbidities or other conditions that make patients poor candidates for surgery, or in the case where the patient refuses any type of surgery.

In 2003, Wang et al. reported the first successful treatment of VPD with topical imiquimod in a patient with recurrent disease [11]. Subsequently, the safety and effec-tiveness of imiquimod was evaluated in two case reports [14,15]. Both (two) patients presented a primary VPD and were treated with topical 5% imiquimod, applied three times a week, for 18 and 25 weeks respectively. The main adverse effect reported was moderate to severe local inflammation; moreover, one patient, reported by Anton et al. [15], experienced two episodes of fever (38 °C) and flu-like symptoms, that did not require treatment interruption. In both cases, the patient showed complete histologic healing with no clinical signs of relapse after a median follow-up of 15 months (12–18 m). Instead, Borrella et al. reported that four out of 55 patients had to stop treatments, due to erosions and local burning in two cases (3%) and in the remaining (3%) cases due to a flu-like syndrome. Of patients who completed treatment, 22 (43%) had a complete response (CR) and 29 (57%) partial response (PR). There were no detected cases of recurrence in patients with a CR also after a prolonged follow-up (mean: 66 months) [39]. In addition, a more recent prospective multicentric study reported that topical treatment with imiquimod was efficacious and safe in patients with non-invasive VPD. Indeed, the response rate was 82.6% in 23 patients: 12 patients (52.2%) showed a CR after 12 weeks of treatment with only four patients (17.4%) having no response. Eight patients developed a recurrence (35%), two within 12 months after treatment while the remaining patients after a median follow-up of 31 months (14–46 months), all of whom had achieved a CR at the end of treatment. Temporary side effects were observed during the treatment; more than 80% of the patients reported pain with three patients (13%) discontinuing the treatment for one week and eight patients (35%) having to reduce the frequency of administration from 3 to 2 times a week due to side-effects [43].

The possibility to combine different types of treatments was already described in 1991 when Ewing reported the successful use of CO_2_ laser after local surgical treatment in six patients with VPD, without recurrences during the follow-up period (ranging from 4– 54 months) [50]. Johnson et al. described the use of laser vaporization, in a case of EMPD extending close to the vaginal mucosa and the urethra, after a wide local excision of the vulva to fully treat the lesion in such delicate areas. Clinical examinations and vulvar biopsy after 6 months of treatment showed no evidence of Paget’s disease [18]. Another type of combined treatment includes fractional CO_2_ laser irradiation followed by photodynamic therapy performed by Ferrara et al., but at the 12-month follow-up only 2/10 (20%) patients had a complete remission, while in two (20%) cases no evident change in the disease status was detectable and in the remaining six (60%) there was a relapse [44]. In a multicentric retrospective study carried out previously, a similar response rate was shown; in fact, CR was reported in 2/13 (15%) patients, furthermore, seven of them (54%) relapsed after a median period of 5 months (1–17 months) [41]. The use of photodynamic therapy in a case of multi-recurrent VPD was described by Vicentini et al., as an alternative to surgery, to be preferred as it is well tolerated by the patient due to the non-invasiveness of the procedure. Complete remission was not achieved, although the patient reported improvement of the symptoms from the first session with a consequent improvement in her quality of life [17].

The role of radiotherapy as an option in the treatment of VPD has not been fully evaluated. Frequently, it is used in case of recurrence in association or as alternative with repeated excisions, particularly in the case of positive margins, dermal invasion, or lymph node metastasis, or as definitive treatment in elderly patients with medical or surgical contraindications. Baiocchi et al. described a case in which after 20 weeks of topical imiquimod treatment, a patient showed a nearly vulvar CR, but after a biopsy on a suspected vaginal area, an invasive Paget disease was found. Then, the patient received conformational external beam radiation therapy (54 Gy), thus achieving a complete clinical and histological response [24].

Surgical excision is considered the gold standard for EMPD, but it is associated with a 30–60% rate of recurrence. To date, the literature is not clear on which surgical technique minimizes local recurrence. As standard surgery, the wide local excision (WLE) has long been considered the standard for the management of VPD, with a surgical margin of 1 to 2 cm. Based on what has been stated so far, several preoperative strategies have been reported in order to reduce the extension of radical surgery without, however, negatively affecting the oncological outcome. In 2013, Kato et al. pro-posed a preoperative biopsy mapping procedure consisting of the removal of 1-cm margin in the case of a well-defined border and margins histologically confirmed and a 3-cm margin in the case of an ill-defined border. They reported residual Paget’s cells in 47% (8/17) of patients at the definitive postoperative histological examination and only one patient (5.9%) with recurrence [51].

A recent preoperative vulvo-vaginal workup tool has been proposed for the prediction of the invasiveness and the extension of VPD called “clock mapping” [6]. Clock mapping consists of multiple vulvovaginal biopsies, carried out in different areas of both the superficial vulvo-perineal area and the central deep vaginal one. First, the surgeon performs biopsies inside the visible lesion, in particular where there is suspicion of an invasive lesion. Then, after drawing a clock outside the visible lesion, in different radial points, the surgeon performs multiple biopsies corresponding to the lesion edges (points A) and at a distance of, respectively, 2 and 4 cm from the lesion borders (points B and C, respectively). Finally, the same procedure is repeated at three vaginal levels for each cardinal point, that is at the “vestibule” and at a distance of 2 and 4 cm from it. This preoperative workup is capable of leading the surgeon in the choice of a better radical surgical strategy according to the final histology of all the clock mapping specimens. In their pilot study, Garganese et al. [10] enrolled 28 women, divided into two groups: 17 (60.7%) in Group A (only intralesional and/or marginal positive biopsies) and 11 (39.3%) in Group B (positive biopsies also beyond the edges of the visible lesion. The clock mapping identified 11 (39.3%) cases with recognized disease extended beyond visible lesion (Group B), allowing personalization of the extent and the shape of resection areas before planning surgical treatment. Moreover, in 23 cases (82.1%), clock mapping identified free surgical margins along the vulvo-perineal skin excision front. On the whole, this technique has emerged as a potentially useful workup tool to predict invasiveness and extension of VPD, in order to tailor surgical excision.

In addition, in order to minimize the radicality of surgery as well as to reduce the recurrence rates, Iavazzo et al. proposed a combined technique using excisional surgery at the edge of 2 cm from the visual lesion borders plus the use of imiquimod in the circumferential area from that limit up to 4 cm around the visual lesion [52].

Further techniques have arisen such as Mohs micrographic surgery (MMS) which provides an intraoperative microscopic assessment of 100% of the tissue margin. Moreover, due to the clinical presentation of VDP, typically as a large and multifocal lesion, MMS can be very costly and time consuming and it can have a similar impact on form and function if compared to WLE when considering this type of lesion [53].

Loiacono et al. observed a high recurrence rate in their retrospective review, of about 33%. Twenty-four patients underwent surgery, particularly 10 (42%), 8 (33%) and 6 (25%) patients underwent extended vulvectomy, simple vulvectomy, and WLE, respectively. However, the recurrence rate was regardless of positive surgical margins, indeed in six cases (25%) surgical margins were not involved [37]. A subsequent retrospective review evaluated treatments and survival outcomes of VPD. Most of the patients (95/122—77%) underwent surgery. Of these, 59 had an intraepithelial VPD, 20 a microinvasive VPD, and 16 an invasive form. Surgical margins were positive in 92% of patients and a local relapse was detected in 73% and, overall, no significant difference was observed among the three groups, regarding the involvement of surgical margins and the risk of recurrence (*p* = 0.33) [38].

## 5. Conclusions

Vulvar Paget’s disease is a chronic disease with a high recurrence rate and low mortality. Since data from this review come from small and retrospective studies, which include both invasive and non-invasive VPD, it is not possible to establish a specific treatment that is the gold standard for all patients, but it should be assessed on a case-by-case basis. However, there were no significant differences in recurrence rates in patients who underwent surgery and those who did not. There also was no association between positive margin status at the time of primary surgery and recurrence.

Surgery may be debilitating, but currently less invasive and destructive techniques are used, such as Mohs micrographic surgery.

Clock mapping can have an important impact on the type of surgery, and it may predict the extension and the invasiveness of the disease beyond visible margins also in the case of an ill-defined border. Regarding the effect on recurrence rates, it can minimize the risk of relapse, as well as the time to recurrence, due to the possibility of obtaining multiple biopsy specimens, if compared to local excision, and increasing the share of obtainable free margins.

Several non-surgical approaches providing both local control of the disease and minimal tissue damage have been developed. Laser therapy or topical treatment with imiquimod may be preferable in patients with initial primary VPD and small and monofocal lesions or in those patients where surgery is not recommended due to comorbidity as well as in elderly women at high anesthetic risk, or in the case of extended and multifocal lesions in which surgery would be excessively destructive. In addition, in more complex and delicate regions to treat, such as the urethra, these treatments should be recommended. Use of topical imiquimod appears to be a therapeutic option both for recurrent and for initial primary vulvar Paget’s disease. Imiquimod may be effective above all in the short term, at least from what can be deduced from the analyzed studies and may be preferable in patients with any type of contraindications to surgery. Furthermore, surgical treatment should be performed in high-end medical centers with dedicated and specialized teams. Lastly, in our review several patients underwent multiple surgical excision or combined treatments, so it is very difficult to clarify whether the long-term recurrence rate after surgery is lower than that with topical imiquimod.

Obviously, a large set of clinical trials needs to be investigated to determine the best medical and surgical strategies in terms of safety and efficacy, while ensuring an adequate quality of life.

In conclusion, as mentioned above, it is difficult to define the criteria for a current therapeutic approach due to the lack of guidelines and the heterogeneity of the patients.

However, to date, due to the different treatment options available and in the absence of a general consensus, it is critical for patient overall survival to tailor treatments to individual patient characteristics and biopsy histopathologic findings, to ensure the best type of therapy.

## Figures and Tables

**Table 1:** Characteristics of the included studies.

Authors	Country	Type of Study	Sample Size, n (Case/Control)	Inclusion Criteria	Age (Years)	Other Neoplasm	Disease STATUS	Symptoms (n–%)	Vulvar Lesion (Before Treatment)	Previous Vulvar Surgery/Medical Therapy n (%)
Hatch et al. (2008) [21]	USA	Case series	2	NA	68 60	None	2 (100%) Relapse	Pain (2–100%) Urinary retention (1–50%)	Erythema	1 (50%) Local resection 1 (50%) Radical vulvectomy
Challenor et al. (2009) [22]	UK	Case series	2	NA	48 66	None	2 (100%) Primary	Itching (2–100%) Soreness (1–50%)	erythema	NR
Sendagorta et al. (2010) [23]	Spain	Case series	3	NA	66 58 82	None	3 (100%) Primary	Pruritis (2–66.6%) Burning (1–33.3%)	Lichenified plaque (1–33.3 %) Erythematous plaque (2–66.6%) Erosions (1–33.3%)	NR
Baiocchi et al. (2012) [24]	Brazil	Case series	4	NA	62.2 (56–80)	None	2 (50%) Primary 2 (50%) Relapse	(4–100%) Pruritis	(4–100%) Erythematous plaque (1–25%) Erosions	(4–100%) Topical antifungals/steroids (1–25%) Simple vulvectomy (1–25%) Wide vulvar resection (1–25%) PDT
Choi et al. (2013) [25]	Korea	Case series	3	- Confirmed VPD	73 (65–81)	NR	3 (100%) Primary	NR	Erythema	treatment
Sanderson et al. (2013) [26]	UK	Case series	6	- Confirmed VPD (primary or recurrent) - Non-invasive	71.5 (58–85)	NR	5 (83.3%) Primary 1 (16.6%) Relapse	Irritation (1–16,6%) Itching (3–50%) Soreness (3–50%) Inflammation (1–16.6%) Painful (1–16.6%)	Plaque	2 (33.2%) Topical steroids 1 (16.6%) Skinning posterior vulvectomy with reconstruction
Cai et al. (2013) [31]	China	Retrospective	43	- Confirmed VPD	68.6 (52–85)	NR	43 (100%) Primary	Pruritis (95.3%) Pain (18.6%) Bleeding (16.3%) Discharge (13.9%)	Erythematous (81.4%) Ulceration (32.6%) Erosion (30.2%) Median size 4.8 cm^2^ (1–10 cm^2^)	(26–61.9%) Topical antifungals/ steroids
De Magnis et al. (2013) [32]	Italy	Retrospective	34	- Confirmed VPD	68.7	5 (14,7%) Breast cancer 2 (5.8%) Vulvar ADC 1 (2.9%) Bladder cancer 1 (2.9%) Lung cancer 1 (2.9%) Basal cell carcinoma 1 (2.9%) Cutaneous Melanoma 1 (2.9%) Vaginal SCC	34 (100%) Primary	Itching (76.5%) Burning (58.8%)	NR	NR
Luyten et al. (2014) [40]	Germany	Retrospective Multicenter	20	- Confirmed VPD - Treated with local imiquimod	66.4 (41–84)	2 (10%) Breast cancers 1 (5%) Malignant tumor of the vulva (Adc, other)	6 (30%) Primary 14 (70%) Relapse	NR	NR	7 (33.3%) Unspecified surgery 3 (14.2%) Laser vaporization 1 (4.7%) Photodynamic therapy
Marchitelli et al. (2014) [27]	Argentina	Case series	10	- Confirmed VPD	71.9 (60–92)	None	7 (70%) Primary 3 (30%) Relapse	NR	Erythematous plaque	3 (30%) NR surgery
Liu et al. (2014) [33]	China	Retrospective	85	NR	64,4 (33–82)	2 (3%) Breast cancer 2 (3%) Cervical cancer 1 (1.5%) Vulvar SCC 1 (1.5%) Rectal cancer 1 (1.5%) Meningioma	85 (100%) Primary	Pruritis (74.1%) Pain (5.9%) Vulvar lesion (20%)	Nonspecific vulvar lesions (17–20%)	NR
Cowan et al. (2016) [42]	USA	Prospective Multicenter	8	- Recurrent VPD - >18	71.5 (47–78)	1 (12.5%) SCC of the face and neck 2 (25%) basal cell carcinoma 2 (25%) Breast cancer	8 (100%) Relapse	Pruritis (5–63%) Burning (2–25%) Pain (2–25%)	Erythema (6–75%)	(6–75%) Simple partial vulvectomies (1–12.5%) Multiple vulvectomies + flaps
Nagai et al. (2016) [28]	Japan	Case series	2	- Confirmed VPD	69 81	NR	2 (100%) Primary	Itching	Eczema	NR
Sopracordevole et al. (2016) [34]	Italy	Retrospective	27	- Confirmed primary VPD - Primary elective surgical treatment - more than 1 y of FUP evaluation	66.5 (36–88)	1 (3.7%) pheochromocytoma 4 (14.8%) breast cancers (bilateral in 2 cases) 1 (3.7%) stomach and colorectal cancer 1 (3.7%) skin carcinoma	27 (100%) Primary	Itching (64.3%) Burning + Itching (14.3%) Pain (14.3%) Burning (7.2%)	Erythemat (73.7%) Erythema + hyperkeratosis (31.6%)	None
Onaiwu et al. (2016) [35]	USA	Retrospective	89	- Confirmed VPD	67 (32–89)	13 (14.6%) Breast cancer 7 (7.9%) Vulvar cancer 6 (6.7%) Bladder cancer 5 (5.6%) Colo-rectal cancer 4 (4.5%) Endometrial cancer	NR	Pruritis (43–48.3%)	NR	NR
Rioli et al. (2018) [41]	France	Retrospective Multicentric	13	- Confirmed VPD - Treated with PDT	70.1 (52–84)	1 (7.6%) Lung Adc	NR Primary NR Relapse	NR	NR	10 (76.9%) Topical imiquimod 8 (61.5%) NR Surgery 6 (46.1) Carbon dioxide laser treatment
Nitecki et al. (2018) [29]	USA	Case series	44	- Confirmed VPD	67 (50–92)	3 (6.8%) Breast cancer 1 (2.2%) Urothelial cancer 1 (2.2%) Lung cancer 2 (4.5%) Cutaneous carcinoma	44 (100%) Primary	Pain (10–22%) Pruritis (10–22%) Pain + pruritus (6–13%)	NR	NR
Nasioudis et al. (2019) [36]	USA	Retrospective	2602	- Confirmed VPD (primary or recurrent)	72 (31–90)	960 (36.9%) NR tumor	NR	NR	Median size 3–4 cm^2^	NR
Loiacono et al. (2019) [37]	Italy	Retrospective	24	- Confirmed VPD	69.3 (38–84)	3 (12.5%) Breast cancer 2 (8%) Endometrial Adc 1 (4%) Vulvar SCC 1 (4%) Ovarian cancer 1 (4%) Melanoma 1 (4%) Adc of the ampulla of Vater 1 (4%) Urothelial carcinoma 1 (4%) Myelodysplastic syndrome	NR	Itching (1–4%) Pruritis (1–4%) Itching + burning + pruritis + vulvar lesions (5–21%) Pain + pruritis (3–13%) Unknown (14–58%)	Median size 48.9 cm^2^ (2.5–143 cm^2^)	NR
Preti et al. (2021) [38]	Italy	Retrospective	122	- Histologically confirmed cutaneous VPD (primary) - at least 6 months follow-up	65 (36–92)	NR	122 (100%) Primary	Itching (59–61%) Burning sensation (18–18%) Itching + Burning (20–21%)	Median size 15 cm^2^ (10–30 cm^2^)	NR
Kosmidis et al. (2021) [30]	Greece	Case series	2	NA	81 69	None	2 (100%) Primary	Pruritis + burning Pruritis + swelling	Erythematous plaque (12.5 × 14.5 cm) Eczematous plaque + erosion (2.4 × 7.8 cm–1 cm × 2 cm)	NR
Ferrara et al. (2021) [44]	Italy	Prospective	10	- Confirmed VPD	79 (67–92)	NR	7 (70%) Primary 3 (30%) Relapse	NR	NR	4 (40%) NR surgery
Borella et al. (2022) [39]	Italy	Retrospective	55	- Confirmed VPD type 1	63 (36–92)	NR	24 (43.6%) Primary 31 (56.3%) Relapse	Itching (29–59%) Burning (15–27%)	Max diameter 60 mm (5–290)	31 (56%) NR surgery
Van der Linden et al. (2022) [43]	The Netherlands	prospective multicentric open-label observational cohort study	24	- Non-invasive VPD - after earlier surgery or imiquimod treatment > 6 months ago - Age ≥ 18 years old	67 (42–84)	NR	20 (83.3%) Primary 4 (16.6%) Relapse	Itching (20–83.3%) Pain (11–45.8%) Burning sensation (17–70.8%) Strangury (7–29.2%) Dyspareunia (5/13–38.5%)	Erythema (24–100%) Scaling (15–62.5%) Ulceration (6–25%) Median size 16 cm^2^ (3–130 cm^2^)	1 (4.1%) Partial vulvectomy 2 (8.2%) NR surgery 1 (4.1%) Vulvectomy 1 (4.1%) Local excision 1 (4.1%) topical 5% imiquimod cream

Adc: adenocarcinoma; NA: not applicable; NR: not reported; PDT: photodynamic therapy; VPD: vulvar Paget’s disease.

**Table 2:** Treatments.

Authors	Medical Treatment	Type of Medical/Other Treatment	Duration of Therapy (Weeks)	Surgical Treatment	Type of Surgical Treatment n (%)	Dermal Invasion
Hatch et al. (2008) [21]	2 (100%)	Topical imiquimod cream 3 times a week Topical imiquimod cream 1 times a day Topical imiquimod cream 2 times a day Topical imiquimod cream 1 times a day Topical 5% imiquimod cream 3 times a week + clobetasol, 0.1% cream	8 w 4 w 2 w 5 w 12 w	None	NA	NR
Challenor et al. (2009) [22]	2 (100%)	Topical imiquimod 5% cream 3 times a week (6 w after surgery) Topical imiquimod 5% cream 3 times a week (8 w after surgery)	12 w 12 w	2 (100%)	1 (50%) Skinning vulvectomy and reconstruction with split skin graft 1 (50%) Skinning vulvectomy with reconstruction by V-Y advancement flaps	NR
Sendagorta et al. (2010) [23]	3 (100%)	Topical imiquimod 5% cream daily Topical 5% imiquimod cream 3 times a week	3 w 3 w	None	NA	NR
Baiocchi et al. (2012) [24]	4 (100%)	(3–75%) Topical 5% imiquimod cream 3 times a week (1–25%) Topical 5% imiquimod cream 2 times a week (1–25%) External RT(54 Gy)	31.5 w (4–52)	1 (25%)	Simple vulvectomy	None
Choi et al. (2013) [25]	3 (100%)	Topical 5% imiquimod cream 3 times a week	24 w	3 (100%)	Local wide excision	None
Sanderson et al. (2013) [26]	6 (100%)	(6–100%) Topical 5% imiquimod cream 3 times a week (3–50%) clobetasone/oxytetracycline/nystatin cream	8–16 w	1 (16.6%)	Vulvectomy	None
Cai et al. (2013) [31]	14 (32.6%)	8 (18.6%) Definitive RT at a median dose 60 Gy + CT 2 (cycle) 6 (14%) adjuvant RT	NR	35 (81.4%)	17 (48.5%) Radical vulvectomy 8 (22.8%) Simple vulvectomy 10 (28.5%) Wide local excision 5 (14.3%) Inguinal LND	7 (16.2%)
De Magnis et al. (2013) [32]	None	NA	NA	34 (100%)	2 (5.9%) Radical vulvectomy1 1 (2.9%) Total simple vulvectomy 10 (29.4%) Partial simple vulvectomy 21 (61.7%) Wide local excision	4 (11.7%)
Luyten et al. (2014) [40]	20 (100%)	Topical 5% imiquimod cream 2 times a week Topical 5% imiquimod cream 3 times a week	15.4 w (4–52)	None	NA	NR
Marchitelli et al. (2014) [27]	10 (100%)	Topical 5% imiquimod cream 3 times a week	22 w (16–28)	None	NA	NR
Liu et al. (2014) [33]	2 (2.9%)	2 (2.9%) RT	NR	69 (81%)	12 (17.4%) Wide local excision 2 (2.9%) Partial vulvectomy 26 (37.6%) Simple vulvectomy 24 (34.7%) Radical vulvectomy 19 (27.5%) Inguinal lymphadenectomy	13 (20′%)
Cowan et al. (2016) [42]	8 (100%)	Topical 5% imiquimod cream 3 times a week	12 w	None	NA	NA
Nagai et al. (2016) [28]	NR	NR	NA	2 (100%)	2 (100%) Wide local excision + split thickness skin graft 1 (50%) LNF dissection	1 (50%)
Sopracordevole et al. (2016) [34]	2 (7.4%)	CO_2_ laser excision	NA	25 (92.5%)	(5–20%) Wide local excision (8–32%) Simple partial vulvectomy (9–36%) Simple total vulvectomy (1–4%) Skinning) total vulvectomy (2–8%) Total vulvectomy with inguino-femoral lymphadenectomy (11–40.7%) Plastic surgery (V-Y plasty, transposition flap, rotational flap, skin graft)	11 (44%)
Onaiwu et al. (2016) [35]	6 (6%)	(4–4.5%) imiquimod (1–1.1%) 5-fluorouracil (1–1.1%) laser ablation	NR	74 (83.1%)	(55–61.8%) Wide local excision (13–14.6%) Radical vulvectomy (4–4.5%) Skinning vulvectomy (2–2.3%) MMS surgery	3 (3.4%)
Rioli et al. (2018) [41]	13 (100%)	Topical 16% methyl aminolevulinate (MAL) + PDT + (1–7.6%) Carbon dioxide laser	NA	None	NA	3 (23%)
Nitecki et al. (2018) [29]	20 (45.4%)	Topical 5% imiquimod cream	NR	42 (95.4%)	(3–7%) MMS surgery (8–19%) Wide local excision (11–26%) Simple partial vulvectomy (17–40%) Radical partial vulvectomy (3–7%) Radical total vulvectomy (6–14%) Reconstructions with advancement flaps (1–2%) Inguinal LNF	12 (27%)
Nasioudis et al. (2019) [36]	156 (5.9%)	(26–0.9%) laser or other ablative methods (35–1.3%%) RT, (72–2.7%) immunotherapy (23–0.8%) C	NR	2412 (92.6%)	(1133–46.9%) Local excision (824–34.1%) Simple/partial vulvectomy (172–7.1%) Total vulvectomy (155–6.4%) Radical vulvectomy (102–4.2%) NR (109–6.8%) LND	1608 (61.8%)
Loiacono et al. (2019) [37]	2 (8%)	(2–8%) Imiquimod and 5-fluorouracil (before surgery)	NR	24 (100%)	(6–25%) Wide local excision (8–33%) Simple vulvectomy (10–42%) Extended vulvectomy (2–8%) LND (7–29%) Reconstructions with advancement flaps	4 (17%)
Preti et al. (2021) [38]	27 (33%)	(13–48%) Topical 5% imiquimod cream (10–37%) Topical corticosteroid cream (4–15%) Laser vaporization	NR	95 (77%)	(41–44%) Local wide excision (26–27%) Hemi-vulvectomy (20–22%) Total vulvectomy (16–13%) Inguinal bilateral LND	16 (16.8%) invasive 20 (21%) Microinvasive
Kosmidis et al. (2021) [30]	None	NA	NA	2 (100%)	Wide local excision + lateral flaps Wide local excision + bilateral flaps	None
Ferrara et al. (2021) [44]	10 (100%)	Fractional CO_2_ Laser irradiation followed by PDT every 2 weeks	5 times 8 w	None	NA	None
Borella et al. (2022) [39]	55 (100%)	31 (56%) Topical 5% imiquimod cream 2 times a week 24 (44%) Topical 5% imiquimod cream 3 times a week	<144 w	None	NA	None
Van der Linden et al. (2022) [43]	24 (100%)	22 (91.7%) Topical 5% imiquimod cream 3 times a week 2 (8.3%) Topical 5% imiquimod cream 3 times a week+/- Topical 3% lidocaine in Vaseline ointment	16 w	None	NA	None

CT: chemotherapy; LND: lymphadenectomy; MMS: Mohs micrographic surgery; NA: not applicable; NR: not reported; PDT: photodynamic therapy.

**Table 3:** Primary and secondary outcomes.

Authors	FUP	Clinical Response n (%)	Vulvar Lesion (After Treatment)	Positive Margin Status	Side Effects (n–%)	OS Median (m)/yrs (%)
Hatch et al. (2008) [21]	12 m 6 m	CR: 2 (100%)	NR	NA	Skin erosion (1–50%) Skin ulceration (1–50%) Hyperpigmentation (1–50%)	NR
Challenor et al. (2009) [22]	4 m 3 m	CR: 2 (100%)	NR	2 (100%) (After surgery)	NR	3.5 m
Sendagorta et al. (2010) [23]	26 m 22 m 20 m	CR: 3 (100%)	NR	NA	Moderate local irritation	NR
Baiocchi et al. (2012) [24]	21 m 40 m NR NR	PR: 1 (25%) CR: 3 (75%) R: 1 (25%)	NR	1 (25%)	Local irritation (4–100%) Local pain (4–100%) Vaginal bleeding (1–25%)	30.5 m
Choi et al. (2013) [25]	38 m (34–46)	CR: 3 (100%)	NR	NR	NR	100% (38 m)
Sanderson et al. (2013) [26]	18 m (12–24)	CR: 3 (50%) R: 1 (16.6%) PD: 2 (33.3%)	NR	NR	(2–33.2%) Soreness (3–50%) Erythema (2–33.2%) Irritation	18 m
Cai et al. (2013) [31]	54 m (7–169)	R:12 (34.3%)	NR	16 (47%)	NR	124.5 m (intraepithelial) 70.8 m (invasive) 21.3 m (adnexal Adc)
De Magnis et al. (2013) [32]	76.9 m (4–184)	R:15 (44.1%) Ç: 1 (2.6%) CR: 26 (76.5%) PR: 1 (2.6%) §: 1 (2.6%)	NR	15 (44.1%)	NR	76.9 m
Luyten et al. (2014) [40]	14.4 m (4–52)	CR: 11 (55%) PR: 5 (25%) SD: 2 (10%) Interruption: 2 (10%)	NR	NR	1 (5%) Local reaction 19 (95%) Well tolerated	14.4 m
Marchitelli et al. (2014) [27]	18.3 m (2–49)	CR: 9 (90%) PR: 1 (10%)	NR	NA	Moderate local irritation Erosion	18.3 m
Liu et al. (2014) [33]	43.6 m	R: 20 (43.5%) after 12.7 m	NR	15 (32.6%)	NR	NR
Cowan et al. (2016) [42]	35 m (5–72)	CR: 6 (75%) after 12 w PR: 2 (25%) R: 4 (67%) L-FUP: 1 (12.5%) Interruption: 1 (12.5%)	NR	NA	Erythema Pain/burning	35 m
Nagai et al. (2016) [28]	12.8 m 112 m	R: 1 (50%) after 2.9 m Ç: 1 (50%) after 12.8 m CR: 1 (50%)	NR	None	NR	112 m
Sopracordevole et al. (2016) [34]	79.5 m (12–313)	R:8 (29.6%)	NR	10 (40%)	NR	NR
Onaiwu et al. (2016) [35]	73.2 m	R: 52 (58.4%) CR: 19 (23.5%) PR: 25 (30.9%) § 45 (55.6%) LFUP 8	No macroscopic EOD	47 (87%)	NR	73.2 m
Rioli et al. (2018) [41]	38 m (4–75)	CR: 2 (15%) PR: 5 (38%) SD: 5 (38%) PD: 1 (8%) R: 7 (54%) after 5 m (1–17)	NR	NA	6 (60%) Moderate/Intense pain	NR
Nitecki et al. (2018) [29]	45.8 m (1–178.9)	R: 25 (56.8%)	NR	43 (97.7%)	NR	28.7 m
Nasioudis et al. (2019) [36]	66.5 m	NR	NR	1214 (58%) 92 NA	NR	84.3% (early-stage disease) 73.6% (advanced stage−81.7% stage II −59.5% stage III −33% stage IV) 53.4% (No surgery) 83.6% (positive surgical margins) 84.6% (negative margins)
Loiacono et al. (2019) [37]	39 m (1−240)	CR: 13 (54%) R: 8 (33%) §: 9 (37.5%) Ç: 1 (4%) LFUP 1 (4%)	NR	12 (50%)	Wound dehiscence (4−17%) Urethral stenosis (4−17%)	39 m
Preti et al. (2021) [38]	94.6 m	R: 69/95 (73%) CR: 79/95 (83.1%) PR: 16/95 (16.8%)	NR	77 (92%) 11 NA	NR	98% (non-invasive and microinvasive VPD) 50% (invasive VPD)
Kosmidis et al. (2021) [30]	NR	NR	NR	None	None	NR
Ferrara et al. (2021) [44]	12 m	CR: 2 (20%) R: 6 (60%) SD: 2 (20%)	NR	NA	Swelling + Pain 2 (20%) Hyperpigmentation	12 m
Borella et al. (2022) [39]	66 m (17−148)	CR: 22 (43%) PR: 29 (56%) Interruption: 4 (7%)	NR	NA	2 (3.6%) Erosion and local burning 2 (3.6%) Flu like syndrome	NR
Van der Linden et al. (2022) [43]	31 m (14−46)	CR: 12 (52.2%) PR: 7 (30.4%) SD: 4 (17.4%) R: 8 (34.8%) LFUP: 1 (4.1%)	1 cm^2^ (0−130 cm^2^; t-test, *p* = 0.001)	NA	(67–71%) Fatigue (17–46%) Headaches (>80%) Pain	31 m

Adc: adenocarcinoma; CR complete response; EOD: evidence of disease; QoL quality of life; PD: progress of disease; PR partial response; SD: stable disease; N/R: not reported R recurrence; L-FUP lost to follow-up; FUP: follow-up; OS: overall survival; NA: not available; 1 patient lost to follow-up; Ç: died of the disease; §: died of other causes.

## Data Availability

Not applicable.
